# Pelvic Pyomyositis in Childhood: Clinical and Radiological Findings in a Tertiary Pediatric Center

**DOI:** 10.3390/children9050685

**Published:** 2022-05-09

**Authors:** Giulia Abbati, Sarah Abu Rumeileh, Anna Perrone, Luisa Galli, Massimo Resti, Sandra Trapani

**Affiliations:** 1 Paediatric Residency, Meyer Children’s University Hospital, Viale Pieraccini 24, 50139 Florence, Italy; sarah.aburumeileh@unifi.it; 2 Department of Health Sciences, University of Florence, Viale Pieraccini 24, 50139 Florence, Italy; luisa.galli@unifi.it (L.G.); sandra.trapani@unifi.it (S.T.); 3 Radiology Unit, Meyer Children’s University Hospital, Viale Pieraccini 24, 50139 Florence, Italy; anna.perrone@meyer.it; 4 Infectious Disease Unit, Meyer Children’s University Hospital, Viale Pieraccini 24, 50139 Florence, Italy; 5 Paediatric Unit, Meyer Children’s University Hospital, Viale Pieraccini 24, 50139 Florence, Italy; massimo.resti@unifi.it

**Keywords:** pyomyositis, muscle infection, abscess, pelvis, pediatric, children, radiology, magnetic resonance imaging (MRI)

## Abstract

Pyomyositis (PM) is an infrequent but increasing bacterial infection of the skeletal muscle, with muscles of the pelvis and thigh frequently involved. The diagnosis is often challenging, especially when a deep muscle is affected. We present a single-center pediatric cohort affected by pelvic PM. A retrospective analysis was performed, including children admitted to Meyer Children’s Hospital between 2010 and 2020. Demographic, anamnestic, clinical, laboratory, radiological and management data were collected. Forty-seven patients (range 8 days–16.5 years, 66% males) were selected. Pain (64%), functional limitations (40%) and fever (38%) were the most common presenting symptoms; 11% developed sepsis. The median time to reach the diagnosis was 5 days (IQR 3–9). Staphylococcus aureus was the most common organism (30%), Methicillin-Resistant S aureus (MRSA) in 14%. PM was associated with osteomyelitis (17%), arthritis (19%) or both (45%). The infection was multifocal in 87% of children and determined abscesses in 44% (40% multiple). Pelvic MRI scan, including diffusion-weighted imaging (DWI), always showed abnormalities when performed. Clinical and laboratory findings in pelvic PM are unspecific, especially in infancy. Nevertheless, the infection may be severe, and the suspicion should be higher. MRI is the most useful radiological technique, and DWI sequence could reveal insidious infections.

## 1. Introduction

Pyomyositis (PM) is an uncommon bacterial infection of the skeletal muscle, which may lead to the occurrence of muscular abscesses and other severe and even life-threatening complications [1,2,3]. Primary PM directly affects the muscle, due to seeding from a transient bacteremia; on the contrary, secondary PM is caused by the extension of a primitive infection of contiguous bones, joints, skin and soft tissues, gastrointestinal tract, urinary tract or other organs, or directly from an open trauma [4,5,6]. PM is classically known to be more frequent in tropical climates [2,4,7,8,9,10], but recently, an increasing incidence has also been reported in subtropical and temperate areas, with several observational studies and case series published in these countries [6,11,12,13,14,15,16,17,18]. All children age groups can be affected, and pediatric patients are often previously healthy [16,19]. Clinical and laboratory features are non-specific, leading to a challenging diagnosis, especially if the infection affects a deep muscle [4,5]. Therefore, an appropriate radiological evaluation is essential, and the technique of choice is magnetic resonance imaging (MRI) [15,20,21,22,23,24]. Muscles of the thigh and the pelvic girdle are mainly involved [4,9,11,14,18]. Symptoms and signs of pelvic PM can be vague at onset, with a high risk of misdiagnosis or delay in diagnosis and treatment and, as a consequence, a greater rate of complications [4,25,26,27]. In fact, despite a high clinical suspicion being recommended, the diagnostic delay is still frequently reported. The purposes of this study are to further define the demographic and clinical data of a cohort of children with acute pelvic PM, to analyze the diagnostic and therapeutic paths applied, and to underline the outcome of these patients admitted to a tertiary-level pediatric hospital. Furthermore, we aimed to determine whether significant differences in age-related presenting manifestations and laboratory abnormalities could be identified.

## 2. Materials and Methods

A retrospective review of all children affected by pelvic PM admitted to the Meyer Children’s University Hospital between January 2010 and December 2020 was carried out. Patients were selected by searching in the institution’s discharge database using the International Classification of Diseases, 9th Revision, Clinical Modification (ICD-9-CM) discharge codes: “infectious myositis”; “muscular abscess”; “muscle infection or necrotizing fasciitis”; “myositis/non-specified myalgia”; “phlegmon”; “phlegmon and abscess”; “gluteal abscess”; “abscess of the lower limb”; “infectious arthritis of the hip”; “pyogenic arthritis of the hip”; “sacroiliitis”; “fasciitis”; “acute osteomyelitis of pelvic bones and femur”; “non-specified osteomyelitis of pelvic bones and femur”; “osteomyelitis of pelvic region and thigh”; “leg osteomyelitis”; and “other infections of pelvic bones and thigh”.

All medical charts were reviewed: patients with a confirmed diagnosis of PM of the pelvic girdle were selected. Pyomyositis, osteomyelitis and arthritis were defined based on imaging features, in addition to clinical presentations. Demographic and anamnestic data including the month of disease onset, predisposing conditions and clinical manifestations (at presentation and during the disease course), time to reach the correct diagnosis from the clinical onset and from hospital admission, initial diagnosis, laboratory results including inflammatory markers, creatine phosphokinase (CK) and etiological researches (on blood and other biological specimens), radiological investigations, muscle involvement (site and abscess formation), treatment (type and duration), length of hospital stay (LOS), follow-up, and outcome have been collected. Patients were then divided into 2 groups (≤6 years; >6 years), in order to analyze the differences in presenting symptoms and laboratory exams.

All the radiological investigations were examined, namely X-ray, ultrasound (US), computed tomography (CT), MRI, bone scintigraphy and ^18^F-fluorodeoxyglucose (FDG) positron emission tomography (PET) scans. MRI has been considered the gold standard for PM diagnosis. The MRI images were revised by an expert pediatric radiologist. MRI was performed using a 1.5T or a 3T scanner (Achieva; Philips Medical Systems, The Netherland). The imaging protocol included spin-echo T1-weighted images (T1W), turbo spin-echo T2-weighted images (T2W), short-tau inversion recovery (STIR) images. A total of 0.2 mmol/kg gadoteric acid (Dotarem; Guerbet, Roissy, France) was administered and a 3-dimensional T1 high-resolution isovolumetric examination (3D-THRIVE) dynamic sequence was acquired. A Diffusion-Weighted Imaging (DWI) sequence (2 b-value: 0, 1000 s/mm^2^) was performed and quantitative analysis of the apparent diffusion coefficient value (ADC) was obtained.

Statistical analysis was performed using SPSS software package (version 20-IBM Analytics). Data were expressed as mean ± standard deviation (SD) or median and interquartile range (IQR). As the distribution of values was Gaussian, parametric tests (*t*-test and ANOVA test) were applied, whereas Pearson’s chi-squared test was employed to determine the differences between the 2 groups of patients. Statistical significance was considered at *p* < 0.05.

## 3. Results

### 3.1. Demographic and Clinical Features

A total of 47 patients with PM were included over an 11-year period. A summary of patients’ demographic characteristics and presenting symptoms is shown in Table S1.

**Figure 1:**
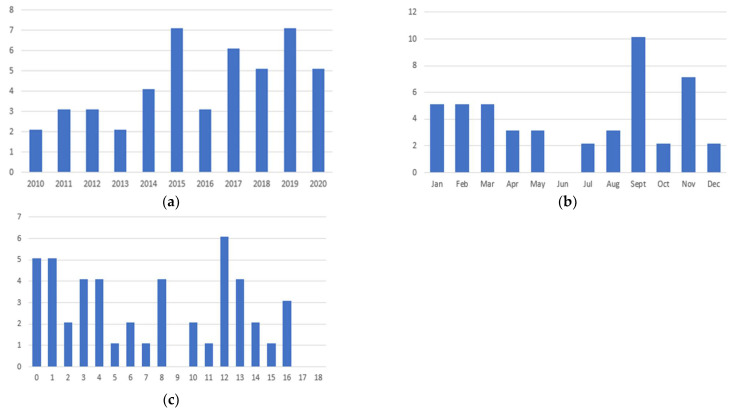
Distribution of cases per year (**a**), per month (**b**) and for age (**c**) in our 47 patients affected by pelvic pyomyositis.

A slight increasing trend in incidence was observed from 2010 to 2020 (Figure 1a), and the majority of cases were observed in September (*n* = 10) and November (*n* = 7), although reported throughout the whole year (Figure 1b).

The age group ranged from 8 days to 16.5 years old, with a median age of 7.5 years (IQR 2.6–12.5) and two peaks of incidence, the first in children aged under 2 years, and the second in adolescents aged 12 and 13 years (Figure 1c); the male to female ratio was 2:1. Most children were previously healthy (*n* = 32), while 15 patients had comorbidities: four had an allergy; three had genetic disorders; obesity was found in two; acute lymphocytic leukemia (ALL) with drug-induced neutropenia; end-stage renal disease (ESRD); antiphospholipid syndrome; eosinophilic gastroenteritis; and celiac disease in one each; finally, a preterm infant had hypogammaglobulinemia. A total of 12 patients (25.5%) reported fever and/or upper respiratory infection in the previous two weeks. A total of 11 children (23.4%) had muscle trauma in the two days prior to presentation, and three of them reported a skin lesion due to such injury. Furthermore, two patients had previously received gluteus intramuscular injection. No patient referred to a history of traveling abroad.

The most common presenting complaints were pain (*n* = 30), fever (*n* = 18), inability to bear weight or other functional limitations (*n* = 19). As shown in Table 1, children older than 6 years complained of pain more frequently than the younger group (*p* = 0.001), whereas younger children tended to present more frequently with irritability (*p* = 0.016). The other manifestations resulted similarly distributed between the two groups.

During the disease course, the majority of patients experienced fever (*n* = 40), inability to bear weight (*n* = 40) and pain, localized at hip (*n* = 33), gluteus (*n* = 16), thigh (*n* = 6) and abdomen (*n* = 4). A total of 26 patients (55.3%) were completely unable to walk. The fever persisted for a median of 5.5 days (IQR 3.3–7.8) and the mean highest temperature was 39 °C (SD 0.8). Five patients (10.6%) developed sepsis during the disease course. On physical examination, 42 patients (89.4%) had pain at the mobilization of the hip, with a limited range of motion, whereas 22 (46.8%) referred to tenderness at the palpation of the muscle. Some children showed cutaneous signs, namely rubor (*n* = 6), calor (*n* = 9), tumor of thigh or gluteus (*n* = 11) and non-specified skin lesions (*n* = 4). One child presented a whitlow at one of the toes, while no patient had a concomitant infection of other apparatuses. The median time from onset to PM diagnosis was 5 days (IQR 3–9), while the median time from hospital admission to diagnosis was 2 days (IQR 1–3.5). There were 13 patients (27.7%) transferred to our ward from other hospitals and 11 (23.4%) were previously discharged from an Emergency Department (ED) at least one time before actual hospitalization. In 20 patients, the first provisional diagnosis was hip septic arthritis, based on Kocher’s criteria; the second most common initial diagnosis was transient synovitis (*n* = 5), found in the younger patients (median age 3.4 years old, IQR 3.3–6.3). Other initial misdiagnoses were osteomyelitis, spondylodiscitis, sacroiliitis, hematoma, Scheuermann disease, neurinoma and pilonidal cyst. Only in five cases (10.6%) the diagnosis of PM was correctly made at the time of hospital admission. The median LOS was 17 days (IQR 10–26). At the time of hospital discharge, no patient presented fever or inability to walk, whereas 20 cases (42.5%) had persistent mild musculoskeletal symptoms, although reduced, such as limp or minimal functional limitation. To note, no patient presented symptoms or signs at the last follow-up.

### 3.2. Laboratory Findings

The main laboratory data are shown in Table S2. ESR, measured in 25 patients on admission, was increased (>31 mm/h) in all of them but two (92%); during hospitalization, the peak mean value was 68 mm/h (SD 28.3). CRP, measured in all patients, was elevated (>0.5 mg/dL) in 45 (95.7%); mean CRP at peak was 10.3 mg/dL (SD 7.2). Analyzing the two age groups and laboratory tests, a positive significant correlation between age and mean CRP at peak was found (Table 1). White blood cell (WBC) count on admission ranged from 8750 to 16,000 cells/µL with a mean value of 12,272 cells/µL (SD 5767). Serum CK, measured in 30 patients, was normal (<200 UI/L) in 28 (93.3%), with a mean value of 108 UI/L (SD 176). Twenty cases (42.6%) had anemia (Hb < 2 SD according to age) with a mean Hb value of 8.75 g/dL (SD 1).

The causative bacterial source was found in 23 patients (48.9%), as shown in Tables S2 and S3. Microorganisms were identified on blood by culture (*n* = 12), *polymerase chain reaction* (*PCR*) technique (*n* = 2) or both (*n* = 2) and on drained abscessual tissue (*n* = 6) by culture (*n* = 1), *PCR* (*n* = 2) or both (*n* = 3). In addition, 12 patients underwent hip aspirate, and in five of them, pathogens were detected by *PCR* (*n* = 4) or both *PCR* and culture (*n* = 1) of the synovial fluid. Overall, the most common isolated organism was Staphylococcus aureus (*n* = 14, 29.8%), found in blood (*n* = 8), drained pus (*n* = 3), blood and synovial fluid (*n* = 2) and synovial fluid alone (*n* = 1). In two cases (14.3%) a Methicillin-Resistant S aureus (MRSA) was found, being one a Panton–Valentine leucocidin (PVL) positive MRSA. Among the septic patients, only one had S aureus positive blood culture; in the other four, S pyogenes, S pneumoniae, P aeruginosa and E faecalis, and F necrophorum were isolated.

### 3.3. Radiologic Investigations

**Figure 2:**
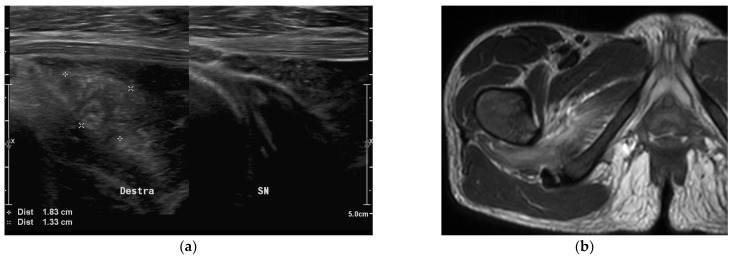
Pyomyositis of the quadratus femoris muscle in a 15-year-old girl affected by end-stage renal disease. (**a**) Soft tissue ultrasound shows enlargement and inhomogeneous hyperechogenic texture of the right muscle, compared to the healthy contralateral muscle. (**b**) Axial T2-weighted MR image shows hyperintense signal in the muscle, no bone or joint involvement.

All patients underwent ultrasound (US) or X-rays or both as first level radiological investigations. Hip and musculoskeletal US were performed, respectively, in 36 (76.6%) and 15 (31.9%) patients, showing muscular involvement in 4 (11.1%) and 11 cases (73.3%). Ultrasound alterations of muscle bundles (usually not homogeneously hyperechoic or anechoic/hypoechoic formations) were described (Figure 2a).

Abdomen or pelvis US were performed in 15 children (31.9%) and were normal in all except one patient, who presented free fluid in the Douglas’ pouch. As for X-rays, 38 patients (80.9%) underwent radiography of pelvic girdle: 11 (28.9%) had pathologic bony or joint involvement, but the muscular infection could not be detected; 4 patients received a radiography of lower limbs and 2 of the lumbar spine. Almost all children (*n* = 45) underwent a pelvic MRI scan; lumbar spine MRI and abdomen MRI were performed in one patient each. CTs were performed in five children: four of them (8.5%) underwent pelvic CT, with description of musculoskeletal involvement only in one case (25%); another child received a lumbar spine CT. Bone scintigraphy was performed only in one child and showed soft tissues involvement. Venous doppler US or electroneurography were each performed once. No patient received ^18^F-FDG PET or PET/CT scans.

The proper diagnosis of PM was established by MRI scan (*n* = 42), CT scan (*n* = 1) or US (*n* = 4). PM was localized on the right side (*n* = 23), on the left side (*n* = 21) or bilaterally (*n* = 2). Localizations of the infection are outlined in Table 2.

Obturator externus was the most frequently affected muscle (*n* = 29), followed by gluteus medius (*n* = 18) and iliacus (*n* = 17). Forty children (85.1%) had a multifocal infection; muscular abscesses were identified in 21 patients (44.7%) and 8 (17%) had multiple abscesses, within the same muscle (*n* = 6) or involving more than one muscle (*n* = 2). One patient had an extra-muscular abscess, localized between obturator externus and pectineus.

**Figure 3:**
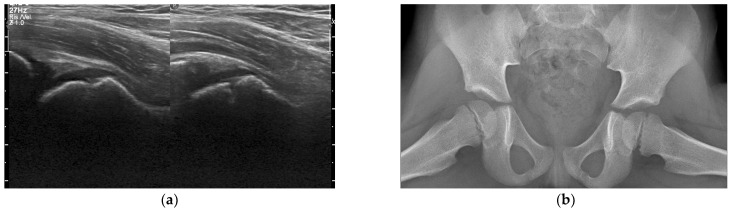

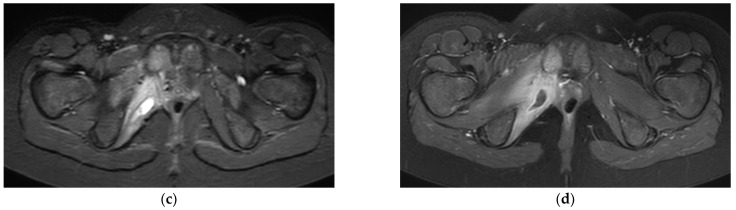
A 5-year-old boy with pyomyositis of the right obturator muscles and osteomyelitis of the right hip bones. (**a**) Soft tissue ultrasound and (**b**) hip X-ray are negative. (**c**) Axial fat suppressed T2-weighted magnetic resonance (MR) image shows muscle edema and an abscess in the obturator internus muscle. On (**d**) fat suppressed axial T1-weighted post-contrast image the abscess shows hypointense fluid collection with rim enhancement. MR images show hyperintensity of the right pubic bone (osteomyelitis).

**Figure 4:**
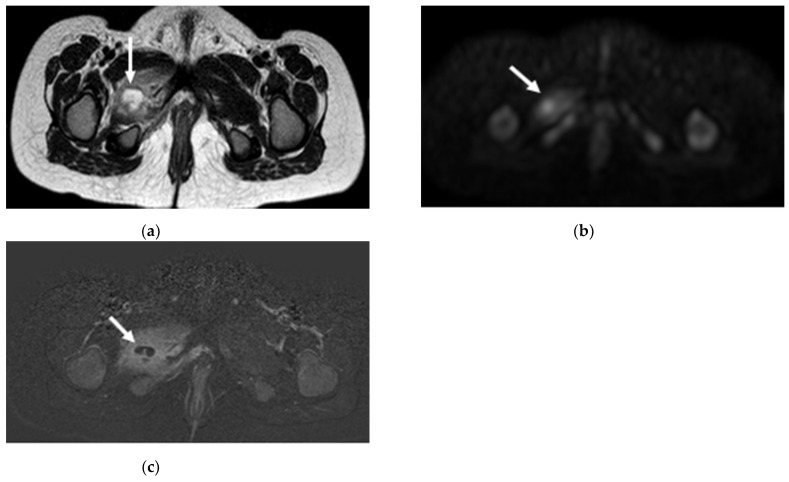
Pyomyositis of the obturator externus muscle in a 3-year-old child with a history of a fall from the bicycle. (**a**) Axial T2-weighted turbo spin echo (TSE) image shows the abscess (arrow) as an hyperintense fluid collection with hypointense wall. The abscess appears hyperintense (arrow) on (**b**) diffusion-weighted imaging (DWI) and hypointense with a thin hyperintense rim (arrow) on (**c**) axial post-contrast image.

MRI showed pathological findings in all children (Figure 2, Figure 3 and Figure 4).

On T1-weighted images, the affected muscles appeared homogeneous (*n* = 34) or heterogeneous (*n* = 7) and they presented an isointense (*n* = 25), hyperintense (*n* = 15) or hypointense (*n* = 1) signal compared to the surrounding healthy muscle. On T2-weighted images, the inflamed muscles showed homogeneous (*n* = 19) or heterogeneous (*n* = 18) signal (Figure 3c and Figure 4a), hyperintense in all but one case (*n* = 36), who presented isointense signal. Even the STIR sequences showed hyperintensity (*n* = 39) or isointensity (*n* = 6). All patients showed post-Gadolinium enhancement when MRI with contrast was performed (*n* = 39) (Figure 3d and Figure 4c). Abscesses appeared homogeneous (*n* = 15) or heterogeneous (*n* = 4) and a peripheric rim was identified in 17 cases with a mean wall thickness of 1.72 mm (SD 0.47; range min 1-max 2.5). A total of 25 patients (55.6%) underwent a DWI sequence: in all these children, the infectious process was evidenced, and abscesses were identified (Figure 4b), even if very small.

In our cohort, 21 patients (44.7%) had both osteomyelitis and arthritis concomitant to muscle infection, 8 patients (17%) only osteomyelitis and 9 patients (19.1%) only arthritis, while the remaining 9 (19.1%) presented PM alone. When affected, the most involved bone and joint were, respectively, the ilium (*n* = 13) and the hip (*n* = 19). Bone marrow edema without osteomyelitis was detected in 4 patients (8.5%). Subcutaneous tissue or fascia adjacent to the infected muscle were involved in 14 patients (29.8%) and 4 (8.5%), respectively.

### 3.4. Treatment

The antibiotic treatment is summarized in Table 3.

Eleven patients (23.4%) began antibiotic therapy before hospitalization. After the admission, all patients received intravenous (IV) antibiotics. Empiric therapy with broad-spectrum antibiotics was started and whenever a specific pathogen was identified, a target therapy was set up. The median total length of IV antibiotic treatment was 18 days (IQR 12–31). In 45 cases (95.7%), antibiotic therapy was switched from IV to oral drugs. The switch was performed when the patient was apyretic, clinically improved and laboratory tests showed decreased inflammatory markers values (negative CRP in 29/39 cases; negative ESR in 14/19 cases) and normalized WBC count (mean 6634 cells/µL, SD 1988). One patient switched to oral therapy after 11 days, but 15 days later he developed a clinical worsening; thus, the IV treatment was restarted and continued for 13 days, with 8 final days of oral regimen. Taking into account the available follow-up data (*n* = 27), oral treatment was administered for a median period of 13 days (IQR 8–15). Eight patients (17%) underwent abscess drainage, through ultrasound-guided percutaneous (*n* = 1), open (*n* = 6) or unspecified (*n* = 1) approach.

### 3.5. Outcome

In 42 cases (89.4%) the infection resolved without any complications. Five children (10.6%) had a complicated course: the patient with ALL under chemotherapy and neutropenia developed sepsis and necrosis of gluteal soft tissues requiring multiple surgical procedures; a 2-week-old-newborn developed sepsis, severe anemia requiring transfusion of red blood cells (RBC) and compartment syndrome undergone to leg fasciotomy; a 2-month-old infant, affected by concomitant femoral osteomyelitis and hip arthritis, developed femoral head dislocation; the adolescent with F. necrophorum PM developed Lemierre’s syndrome and septic shock; a 6-week-old, preterm infant presented severe anemia due to the deep infection and RBC transfusion was necessary.

Based on the available data, 21 patients (44.7%) had a clinical follow-up; no patient had relapse or short-term complications at a median follow-up of 31 days (IQR 12.25–75.75). Twenty-two patients (46.8%) underwent a radiological follow-up using at least one imaging examination (US, X-ray and/or MRI) and 18 (38.3%) received a radiological follow-up by MRI.

## 4. Discussion

Pyomyositis is a severe bacterial infection within the skeletal muscle. First described in 1885 as an endemic disease in the tropical areas [7], it is still more frequent in the tropics [2,4,8,9,10]. However, several cases of PM have been reported even from subtropical and temperate countries, where it seems to have an increasing incidence [16,28]. This trend was confirmed even in our cohort since we observed a slight increase of patients affected by PM from 2010 to 2020 (Figure 1a). Nevertheless, we found in the literature only a few pediatric series, mostly case reports, reported in Italy [19,27,29,30,31,32].

Based on the pathogenesis, PM can be considered primary or secondary. In our cohort, both patients affected by isolated PM and those with PM concurrent to osteomyelitis and/or arthritis have been included [33]. Some authors suggest that when PM is concomitant to bone and/or joint infection, it should be considered secondary, given the intrinsic resistance of the muscle to the infection [23,34,35]; on the other hand, osteomyelitis and arthritis are reported to be common complications of PM, especially in cases with late diagnosis [36,37,38]. In the study of Aderele et al., 25% of children with PM had associated osteomyelitis, with a high incidence of complications and associated disorders [38]. In our study, only 20% of children presented PM alone, without osteomyelitis or arthritis. In addition, frequent MRI findings in patients with PM are reactive joint effusions or signal alterations of a nearby bone, and differential diagnosis between osteomyelitis and reactive bone marrow edema is not simple, even after contrast administration [24,39,40].

Primary muscle infection is rare, and the infection is probably a complication of transient bacteremia, because it develops without a clear entry site in most patients [11]; the concomitant alterations of the muscle structure seem to play a significant role [4,5,6]. Even if the role of trauma in pathogenesis is controversial [4,9], a preceding trauma is reported by many authors [14,23,27,41]. Even in our series, about a quarter of children referred a muscle trauma, mainly a fall, and two had received gluteal intramuscular injection before clinical onset. In the remaining patients, a previous trauma was denied, but still, an occult/neglected trauma or history of intense exercise are probable events in children [30,41,42,43]. Other predisposing factors reported in the literature are immunodeficiency, malnutrition, vitamin deficiencies, chronic illness such as diabetes mellitus or chronic renal failure, penetrating trauma, parasitic or viral infections [6,14,22,44,45]. In our series, only two patients had immunological alterations, but most of our children were healthy, as expected in childhood [16,27,32], in contrast to adult patients [4,41]. One child presented a whitlow at one toe together with multifocal PM and iliacus abscess, osteomyelitis and arthritis; few cases of PM preceded by a whitlow are described in the literature [46].

The literature shows that pediatric cases of PM can occur in all the ages; some authors found it mostly in children within the first decade of life [12,47], with a peak between 2 and 5 years old [9], while others reported that pelvic PM might affect more often teenagers [17]. In our cohort, children of almost all the ages were affected, ranging from 8 days to 16.5 years old, but age distribution was heterogeneous, with two peaks of incidence: the first in children <5 years old (even more <2) and the second in preadolescents aged 12 and 13 years (Figure 1c). Noteworthy, even neonatal cases of pelvic PM were reported [19]; two newborns were included in our series, both with a complicated course. Males were predominantly affected in our series, in agreement with the literature [9,14,28,48].

PM more frequently affects large muscle masses such as the thigh and pelvis, although every muscle can be involved [4,9,11,14,18]; in our series, obturator externus was the most frequently affected muscle, alone or in association, and the most common site of abscesses. A multifocal infection was reported in the literature in 15–43% of cases [9,37]; in contrast, a higher percentage (85%) of our patients had more than one muscle involved.

The diagnosis of PM is often challenging, especially when a deep muscle is affected, because of the non-specificity of clinical presentation and laboratory markers and low suspicion by clinicians. Furthermore, the wide clinical spectrum depends on the frequent association with adjacent osteomyelitis or arthritis. As reported [18], septic arthritis is the main differential diagnosis and many of our patients initially fulfilled Kocher’s criteria. As a result, the time to reach the correct diagnosis may be prolonged. Actually, the mean delay between clinical onset and diagnosis was about 5 days in our cohort, and a previous evaluation by a primary care pediatrician or at the ED was seldom performed. This finding is consistent with other studies [17] and Bickels et al. even described a mean delay of 10 days in their review [4].

Clinical features in our series were similar to those previously described in the literature [11,14,17,22,28]. Pain, functional limitation and fever accounted for the most common initial complaints. Although about 85% of our patients developed fever during the disease course, only 38% were feverish on clinical onset. An insidious challenge for the clinician is to diagnose PM in younger patients, who are often unable to verbalize pain and often present vague symptoms such as irritability, reduced activity, decreased range of motion or inability to bear weight [49]. In fact, we found a significant correlation between age and symptoms at onset, being pain more significantly recorded in children older than 6 years old, and irritability in the younger group.

In our study, no laboratory tests were proven to be specific for PM, although elevated CRP and ESR were frequently found, accordingly to the literature [11,16]. Unnikrishnan et al. assumed that primary PM was unlikely without inflammatory marker elevation [16]. Our study showed a significant positive correlation between age and the mean CRP value at peak, resulting in significantly higher in the older group. Elevated CK was extremely rare in our patients, in agreement with other series, and this finding can help in the differential diagnosis between PM and viral myositis or polymyositis, where CK was generally increased [14,37,50]. Anemia is already reported in the literature [38] and was frequently found in our study group (42%), although five children had other concomitant predisposing conditions, namely ALL, hematochezia in eosinophilic gastroenteritis, ESRD, prematurity, and thalassemic trait.

The gold standard for etiological diagnosis should include a surgical exploration to obtain muscle histology and microbiological investigations [6]; however, this invasive procedure is not frequently performed, especially in pediatric age. Overall, the bacteria causing PM are not always easily identifiable neither on blood nor on pus [4,23]. Accordingly, we identified the responsible bacterium in almost 50% of cases, in line with other studies [14,36]. S aureus is the most common etiological agent found in up to 90% of cases [4,14,15,16,17,28,41,48], and it was detected in 30% of all our patients and in 60% of those with identified microorganisms; MRSA was isolated in two children, representing nearly 15% of cases, and PVL positivity in one. These data agree with previous studies [17,18], although recent reports showed worldwide an increasing prevalence of community acquired-MRSA (CA-MRSA) in PM in up to 64% of the isolated S aureus [28,37,50]. Some authors referred that the infections caused by MRSA and PVL-positive S aureus were associated with larger abscesses, a prolonged LOS and a higher rate of complications [37]; conversely, the disease course of our two MRSA patients did not allow to confirm these correlations. Other studies reported S pyogenes and, less frequently, E coli, S pneumoniae, Klebsiella or further organisms involved in PM [11,15,36,48,51,52]. Notable, P aeruginosa, a rare causative pathogen, was isolated from our patient affected by ALL, as recently reported in another paper [31]. In our series an immunocompetent teenager developed multifocal PM caused by F necrophorum and complicated by Lemierre’s syndrome; this presentation, already described in the literature [53,54], was nevertheless extremely uncommon. Group Y N meningitidis in the synovial fluid was detected in one patient affected by hip septic arthritis and PM of the iliacus muscle; in the literature, a few cases of meningococcal pyomyositis (group B N meningitidis) were described [55,56] but none with meningococcal Y.

PM presents three clinical stages: “invasive,” “purulent” and “late stage” [9,50,57]. Muscular abscesses, common features of PM, develop in the purulent stage, which occurs 10–21 days after the onset; Chiedozi et al. reported that more than 90% of patients were admitted in this stage [9]; in addition, multiple abscesses were reported in 15–40% of cases [9,58]. In our cohort, abscesses were present in less than half of our patients, and multiple abscesses in 17%, assuming that our patients were quite early seen and diagnosed; our median time to diagnosis of 5 days should be considered reasonably short. The third phase (“late stage”) was characterized by systemic clinical features and, if prompt treatment is not established, severe complications, such as compartment syndrome, septic shock and even death, can occur [4,9,40,59]. Five of our patients developed at least one complication, but no one died or had sequelae at the last follow-up.

To avoid diagnostic delay and severe complications, a correct diagnostic approach with appropriate imaging is paramount [27]. MRI represents the technique of choice as it allows to early detect muscle infection, even in the invasive stage, and to define the extension of disease and the involvement of contiguous structures [6,22,23,60,61,62]. CT scan may document muscle infection and fluid collection and it plays a role in guiding drainage of deep-seated abscesses; however, MRI is preferred to CT for better soft-tissue contrast and radiation sparing [39,63]. Bone scintigraphy is a sensitive technique to reveal bone and muscle infections, but its use is limited to selected cases [23]. ^18^F-FDG PET/CT scan, providing combined metabolic and anatomical information, may play a role in detecting soft-tissue, musculoskeletal and abdominal infections or abscesses [64]. The use of PET/CT is limited in the pediatric age due to the high radiation dose; pediatric oncology remains the main application field, but its use for infectious/inflammatory musculoskeletal disorders is increasing [65,66]. Furthermore, it allows a whole-body investigation revealing the sites and extensions of disseminated infections, including PM, in one examination [67]. US is also used to study the soft tissues; the infected muscle is described as having homogeneous or inhomogeneous hyperechogenic texture, with hypoechogenic areas in case of abscesses [33,68]; our US results are in line with these findings. US can be used to grossly but rapidly localize an inflammatory process and to guide the aspiration of an abscess when situated in a superficial muscle [23]. Gubbay et al. obtained a diagnostic US study in 6 out of 13 patients [14]; Trusen et al. reported that US was not conclusive in 3 out of 4 patients with pelvic PM, whereas it resulted in diagnostic in all 8 cases of PM of the extremities [33]. In our study, hip US showed muscle involvement only in a small percentage (10%) of patients who underwent the imaging, but the musculoskeletal US highlighted muscle involvement in about 75% of cases. Therefore, US could be the first imaging approach even in the pelvis, especially when the involvement of a superficial muscle is evident by local signs or symptoms of inflammation. When US findings are consistent with PM, the diagnosis could be confirmed, and MRI can be performed later. On the other hand, if US is negative, PM cannot be excluded because altered echogenicity and deep intramuscular fluid collections can be lost [22,33], and MRI should always be performed. We highlight that, among our 12 patients with US muscle abnormalities, only four received an echographic diagnosis of PM, and only two did not perform further investigations, as US was considered sufficient due to the superficial localization of the infection.

Characteristic MRI findings have been well described [22,23,24,39]; the infected muscles appear enlarged and swollen and they usually show homogeneous or heterogeneous hyperintense signal on T2WI and STIR sequences and intermediate to slightly increased signal intensity on T1WI. Our results largely agree with these descriptions. In presence of an abscess, a very high signal is evident on T2 or STIR sequences; however, small abscesses cannot be appreciated on pre-contrast images, whereas they are identified as fluid signal cavities with peripheral rim enhancement after contrast agent administration [24]. In addition, DWI sequence allows revealing an abscess as a cavity with central restricted diffusion (low value of ADC), surrounded by muscle edema, which shows increased diffusion due to inflammation [69,70]. In our series, when DWI sequence was performed, it was able to identify also very small abscesses. Therefore, the use of gadolinium should be selected case-by-case; its sparing would have worthy advantages as reducing costs, time and need for anesthesia.

Treatment of PM includes early IV antibiotic therapy and abscess drainage by open or percutaneous procedures, where appropriate. In our series eight patients underwent abscess drainage, mostly by the open approach, even if Palacio et al. reported that the percutaneous approach showed efficient results, with a shorter length of antibiotic administration and hospital stay [71]. Antibiotic treatment should be tailored to the specific etiological agent, when identified. Because S aureus is the most common causative organism, in the empirical antibiotic regimen a penicillinase-resistant penicillin should be used; a coverage for CA-MRSA should be established in countries with a high proportion of this isolate; clindamycin with or without penicillin resulted effective and even superior to penicillin alone [50]. Alternative options in the case of MRSA are vancomycin, teicoplanin or linezolid. The most used empiric regimen (57%) in our series was a combination of a Beta-Lactamase Resistant Penicillin with a third-generation cephalosporin. Only two patients received clindamycin at the beginning of treatment, whereas it was added subsequently in other 18 patients. IV treatment is recommended until a clinical and laboratory improvement has been reached; nevertheless, in our study IV antibiotics were administered for a longer period (median of 18 days) compared to other series [6,37]. Treatment was then continued orally for a median of 13 days, in accordance with the literature [37,50]. Overall, treatment duration is not standardized. Pannaraj et al. postulated that three weeks of treatment seems appropriate when bone and joint are not involved [37]. Treatment in our study resulted heterogeneous in type and duration: partial explanations are that many patients presented concomitant joint and/or bone infection and that in about one-third of the cases, an empiric treatment was chosen by the referring hospital prior to the transfer of the patient.

## 5. Conclusions

Pyomyositis is a rare infection, but its incidence is apparently increasing, also in non-tropical climates. Muscular infection is often associated with adjacent osteomyelitis and/or arthritis. The disease can be severe, with a frequent occurrence of abscesses and multifocal involvement and possible complications, including septic evolution. The diagnosis of pelvic PM can be late because of the low suspicion by pediatricians and because the clinical presentation and laboratory exams are non-specific, especially in young children. Infants represent the most insidious patients as they may not express pain, not have significantly high CRP values and develop complications such as sepsis in a higher proportion of cases. MRI is the preferred radiological technique, especially for the pelvis, and DWI sequence could represent an important tool to detect even insidious infections, such as small abscesses, sparing gadolinium administration. Prompt IV antibiotic treatment targeted on S aureus is generally required, and coverage for CA-MRSA should be considered case by case in Italy.

## Figures and Tables

**Table 1:** Comparison of presenting symptoms and laboratory tests in the two different age groups.

	≤6 Years Old (*n* = 23)	>6 Years Old (*n* = 24)	
Presenting Symptoms	Value		*p*
Fever, *n* (%)	11 (47.8)	7 (29)	0.188
Pain, *n* (%)	9 (39.1)	21 (87.5)	**0.001**
Inability to weight bear and/or functional limitation, *n* (%)	10 (43.5)	9 (37.5)	0.67
Limp, *n* (%)	5 (21.7)	4 (16.7)	0.659
Skin alterations, *n* (%)	2 (8.7)	0	0.140
Localized swelling, *n* (%)	2 (8.7)	3 (12.5)	0.672
Irritability, *n* (%)	5 (21.7)	0	**0.016**
**Laboratory Tests**	**Value**		***p***
Serum CRP, mean (SD)—mg/dL	7.6 (5.6)	13.2 (7.5)	**0.007**
ESR, mean (SD)—mm/h	61.1 (27.1)	72.1 (27.3)	0.332
Serum PCT, mean (SD)—mg/dL	6.3 (4.17)	6.7	0.956

CRP: C-reactive protein; ESR: erythrocyte sedimentation rate; PCT: procalcitonin.

**Table 2:** Pelvic girdle involvement in our 47 patients affected by pyomyositis.

Involved Muscles	Pyomyositis, No. (%) (*n* = 47)	Abscesses, No. (%) (*n* = 21)
Obturator externus	29 (61.7)	7 (14.9)
Obturator internus	15 (31.9)	3 (6.4)
Iliacus	17 (36.2)	4 (8.5)
Psoas	6 (12.8)	3 (6.4)
Pectineus	9 (19.1)	2 (4.3)
Piriformis	10 (21.3)	1 (2.1)
Iliocostalis lumborum	2 (4.3)	/
Gluteus maximus	10 (21.3)	4 (8.5)
Gluteus medius	18 (38.3)	1 (2.1)
Gluteus minimus	15 (31.9)	/
Adductor magnus	4 (8.5)	2 (4.3)
Adductor longus	2 (4.3)	1 (2.1)
Adductor brevis	3 (6.4)	1 (2.1)
Gemelli muscles	2 (4.3)	/
Quadratus femoris	8 (17)	1 (2.1)
Quadratus lumborum	1 (2.1)	/
Rectus femoris	1 (2.1)	/
Vastus lateralis	2 (4.3)	1 (2.1)
Tensor fasciae latae	2 (4.3)	/
Quadriceps femoris	1 (2.1)	/
Others than pelvis	Thigh 3 (6.4) Paravertebral muscles 2 (4.3)	/ /

**Table 3:** Oral and intravenous antibiotic treatment in our 47 patients affected by pyomyositis.

Antibiotics	Oral Drugs before Hospitalization, No. (*n* = 11)	Intravenous Drugs, No. (*n* = 47)	Switch to Oral Drugs, No. (*n* = 45)
Amoxicillin or flucloxacillin			5
Oxacillin		30	
Amoxicillin-clavulanate	8		22
Ampicillin-sulbactam		2	
Piperacillin-tazobactam		1	
1st generation cephalosporin (cephalexin, cefazolin)		3	1
Cefpodoxime	2		
Ceftriaxone		24	
Ceftazidime		11	
Aminoglycosides		11	
Clindamycin		20	9
Teicoplanin		15	
Vancomycin		2	
Carbapenems		6	
Linezolid		3	2
Tigecycline		3	
Rifampicin			10
Cotrimoxazole			6
Others *	1	8	2

* clarithromycin, fluoroquinolones, metronidazole, sodium and potassium penicillin.

## Data Availability

The data presented in this study are available on request from the corresponding author.

## Supplementary Materials

The following supporting information can be downloaded at: https://www.mdpi.com/article/10.3390/children9050685/s1, Table S1: main demographic and clinical data of our 47 patients affected by pelvic pyomyositis; Table S2: main laboratory findings of our 47 patients affected by pelvic pyomyositis; Table S3: Microbiological findings in the 23 patients with identified causative pathogens.
