# Supplementary material for: Pelvic Pyomyositis in Childhood: Clinical and Radiological Findings in a Tertiary Pediatric Center
Source: Children (Basel). 2022 May 9;9(5):685. doi: 10.3390/children9050685 (PMC9139856; doi:10.3390/children9050685)
Supplement: Supplementary file 1 [file children-09-00685-s001.zip › Table S1.pdf]

Table S1. Main demographic and clinical data of our 47 patients affected by pelvic pyomyositis.

Characteristics	Value
Age at diagnosis, median (IQR) - *years*	7.5 (2.6-12.5)
Gender, n (%)	
Male	31 (65.9)
Nationality, n (%)	
Italian	40 (85.1)
Others	7 (14.9)
Time to diagnosis, median (IQR) - *days*	5 (3-9)
LOS*, median (IQR) - *days*	17 (10-26)
**Presenting symptoms**	**Value**
Fever, n (%)	18 (38.3)
Pain, n (%)	30 (63.8)
Inability to weight bear and/or functional limitation, n (%)	19 (40.4)
Limp, n (%)	9 (19.2)
Skin alterations, n (%)	2 (4.2)
Localized swelling, n (%)	5 (10.6)
Irritability, n (%)	5 (10.6)
**Symptoms during the disease course**	**Value**
Fever, n (%)	40 (85.1)
Fever duration, median (IQR) - *days*	4.5 (2-7.25)
Sepsis, n (%)	5 (10.6)
Inability to weight bear, n (%)	40 (85.1)
Hip pain, n (%)	33 (70.2)
Gluteus pain, n (%)	16 (34)
Thigh pain, n (%)	6 (12.8)
Abdominal pain, n (%)	4 (8.5)
Localized swelling, n (%)	11 (23.4)
Skin alterations, n (%): hyperaemia	6 (12.8)
calor	9 (19.1)
skin lesions	4 (8.5)

*LOS: length of hospital stay.
