# Supplementary material for: Pelvic Pyomyositis in Childhood: Clinical and Radiological Findings in a Tertiary Pediatric Center
Source: Children (Basel). 2022 May 9;9(5):685. doi: 10.3390/children9050685 (PMC9139856; doi:10.3390/children9050685)
Supplement: Supplementary file 1 [file children-09-00685-s001.zip › Table S2.pdf]

Table S2. Main laboratory findings of our 47 patients affected by pelvic pyomyositis.

**Laboratory tests**	**Value**
Serum CRP, mean (SD) - *mg/dL*	10.3 (7.2)
Serum PCT, mean (SD) - *ng/mL*	3.5 (3.2)
ESR, mean (SD) - *mm/h*	68 (28.3)
Serum WBC count, mean (SD) - *cells/µL*	12,272 (5,767)
Hb, mean (SD) - *g/dL*	10.6 (2.4)
**Cultured microorganism**	**No.**
Blood	14 (10 S aureus, 1 S pneumoniae, 1 S pyogenes, 1 S agalatiae, 1 P aeruginosa and E faecalis)
Drained pus	4 (3 S aureus, 1 P aeruginosa and E faecalis)
Synovial fluid	1 (1 S aureus)
**Microorganism *PCR***	**No**.
Blood	4 (2 S pyogenes, 2 S aureus)
Drained pus	5 (3 S aureus, 1 S pneumoniae, 1 F necrophorum)
Synovial fluid	5 (2 S aureus, 1 S aureus and S pyogenes, 1 S agalatiae, 1 group Y N meningitidis)

CRP: C-reactive protein; PCT: procalcitonin; ESR: erythrocyte sedimentation rate; WBC: white blood cells; Hb: haemoglobin; *PCR*: *polymerase chain reaction*.
