# Supplementary material for: Pelvic Pyomyositis in Childhood: Clinical and Radiological Findings in a Tertiary Pediatric Center
Source: Children (Basel). 2022 May 9;9(5):685. doi: 10.3390/children9050685 (PMC9139856; doi:10.3390/children9050685)
Supplement: Supplementary file 1 [file children-09-00685-s001.zip › Table S3.pdf]

Table S3. Microbiological findings in the 23 patients with identified causative pathogens.

Case No.	Age (years)	Blood		Muscle drainage		Synovial fluid		Complications
		**Culture**	***PCR***	**Culture**	***PCR***	**Culture**	***PCR***	
1	12.70	S aureus	-	NP	NP	NP	NP	
2	0.04	S pneumoniae	-	-	S pneumoniae	NP	NP	Sepsis, severe anaemia, compartment syndrome
3	10.97	S aureus	-	NP	NP	NP	NP	
4	12.45	S aureus	NP	NP	NP	-	S aureus	
5	8.43	S aureus	-	NP	NP	-	-	
6	2.05	P aeruginosa E faecalis	NP	P aeruginosa E faecalis	NP	NP	NP	Sepsis, soft tissues necrosis
7	13.99	MRSA	S aureus	NP	NP	-	-	
8	13.60	S aureus	S aureus	NP	NP	S aureus	S aureus	
9	3.33	S aureus	-	NP	NP	NP	NP	
10	0.13	S agalactiae	-	NP	NP	NP	NP	Severe anaemia
11	16.28	S aureus	-	NP	NP	NP	NP	Sepsis
12	11.65	S pyogenes	-	NP	NP	-	-	Sepsis
13	12.19	S aureus	-	NP	NP	NP	NP	
14	13.32	S aureus	-	NP	NP	NP	NP	
15	4.81	-	S pyogenes	NP	NP	NP	NP	
16	6.27	-	S pyogenes	NP	NP	-	-	
17	0.02	NP	NP	MRSA PVL+	S aureus	NP	NP	
18	8.13	-	-	S aureus	S aureus	NP	NP	
19	16.18	NP	NP	S aureus	S aureus	NP	NP	
20	16.51	-	-	-	F necrophorum	-	-	Septic shock, Lemierre's syndrome
21	8.97	-	-	NP	NP	-	group Y N meningitidis	
22	7.48	-	-	-	-	-	S aureus S pyogenes	
23	0.17	-	-	-	NP	-	S agalactiae	Femoral head dislocation

PCR: *polymerase chain reaction*; -: negative; NP: not performed; MRSA: Methicillin-Resistant S aureus; PVL: Panton-Valentine leucocidin.
